# Potentiation of NETs release is novel characteristic of TREM-1 activation and the pharmacological inhibition of TREM-1 could prevent from the deleterious consequences of NETs release in sepsis

**DOI:** 10.1038/s41423-020-00591-7

**Published:** 2021-01-08

**Authors:** Amir Boufenzer, Kevin Carrasco, Lucie Jolly, Benjamin Brustolin, Elisa Di-Pillo, Marc Derive, Sébastien Gibot

**Affiliations:** 1 INOTREM, Vandœuvre-lès-Nancy, Nancy, France; 2 UMR-S 1116, Défaillance Cardiovasculaire Aigue et Chronique, Vandœuvre-lès-Nancy, Nancy, France; 3 CHRU Nancy, Hôpital Central, Service de Réanimation Médicale, Nancy, France

**Keywords:** TREM-1, NETs, sepsis, endothelial cell activation, vascular dysfunction, LR12, Cell death and immune response, Sepsis

## Abstract

During sepsis, neutrophil activation induces endothelial cell (EC) dysfunction partly through neutrophil extracellular trap (NET) release. The triggering receptor expressed on myeloid cell-1 (TREM-1) is an orphan immune receptor that amplifies the inflammatory response mediated by Toll-like receptor-4 (TLR4) engagement. Although the key role of TLR4 signaling in NETosis is known, the role of TREM-1 in this process has not yet been investigated. Here, we report that TREM-1 potentiates NET release by human and murine neutrophils and is a component of the NET structure. In contrast, pharmacologic inhibition or genetic ablation of TREM-1 decreased NETosis in vitro and during experimental septic shock in vivo. Moreover, isolated NETs were able to activate ECs and impair vascular reactivity, and these deleterious effects were dampened by TREM-1 inhibition. TREM-1 may, therefore, constitute a new therapeutic target to prevent NETosis and associated endothelial dysfunction.

## Introduction

Sepsis is defined as life-threatening organ dysfunction caused by a dysregulated host response to an infection and remains a leading cause of death worldwide.^1^ The host response involves neutrophil activation and neutrophil-mediated compromise of endothelial barrier integrity, which contribute to end-organ dysfunction.^2^

The primary responses of neutrophils to invading microbes are the formation of reactive oxygen species (ROS), the release of cytokines, and the engulfment of pathogens into phagosomes.^3^ Activated neutrophils can also expel nuclear DNA associated with histones and proteins, which form extracellular web-like structures called neutrophil extracellular traps (NETs).^4^ NET release (NETosis) is an active process that is distinct from apoptosis or necrosis.^5^ The mechanism by which neutrophils form NETs depends on the nature of the stimulus.^6^ While these stimuli engage diverse pathways, they all converge to a common outcome characterized by histone modification, chromatin decondensation, the loss of the nuclear envelope, the mixing of nuclear contents and cytoplasmic granular proteins, the loss of membrane integrity, and ultimately, the release of neutrophil extracellular structures.^6^ As such, NETs may function as a double-edged sword: on the one hand, NETs constitute an effective antimicrobial defense^4^; on the other hand, they are a source of molecules with immune effector and proinflammatory functions that contribute to the pathophysiology of septic shock.^7–9^ One of the hallmarks of sepsis is endothelial dysfunction,^2^ which leads to dysregulated hemostasis and vascular reactivity,^10^ and the amplification of the inflammatory response is associated with adhesion molecule overexpression, increased leukocyte trafficking, and excessive NET formation.^11^ In particular, NETs and their components, especially histones, may amplify the inflammatory process,^11,12^ induce endothelial cell activation,^13^ promote endothelial barrier dysfunction through the disruption of adherens junctions, and may thus mediate microvascular leakage.^14–20^ In addition, circulating cell-free DNA, myeloperoxidase-DNA (MPO-DNA), and citrullinated histone H3 (Cit-H3) have been shown to correlate with the severity of organ dysfunction and 28-day mortality in septic shock patients.^15–18^

The immune receptor triggering receptor expressed on myeloid cell-1 (TREM-1) plays a crucial role in the amplification of the inflammatory response, partnering with other pattern recognition receptors such as Toll-like receptors (TLRs).^19^ TREM-1 expression has been observed in different cell types, such as neutrophils, mature monocytes, macrophages, natural killer cells, platelets, and endothelial cells.^20–22^ The structure of TREM-1 includes three distinct domains: an immunoglobulin (Ig)-like structure, a transmembrane part and a short cytoplasmic tail. For signal transduction, TREM-1 associates with immunoreceptor tyrosine-based activation motif-containing adaptor protein (DAP12) through an electrostatic interaction between a negatively charged (−) aspartic acid in DAP12 and a positively charged (+) lysine within the transmembrane domains.^20,23^ TREM-1 engagement following TLR activation ultimately leads to amplification of the inflammatory response.^24^

Fortin et al.^25^ showed for the first time that after TLR4 activation, TREM-1 and TLR4 colocalize into GM1 lipid rafts, a phenomenon that was thought to facilitate the assembly of extracellular and intracellular TLR4 and TREM-1 pathway mediators. This assembly results in the phosphorylation of diverse signaling molecules (Lyn, AKT, extracellular signal-regulated kinase (ERK1/2), and Jak2) and the induction of ROS.^25^ These findings have been further confirmed in several studies,^26,27^ and some of these intracellular pathways have been further shown to play important roles in NETosis.^28–30^

We recently showed that the pharmacological inhibition of TREM-1 (with the synthetic peptide LR12) or the genetic ablation of TREM-1 in endothelial cells reduced neutrophil infiltration, vascular endothelial dysfunction, and death in various experimental septic shock models.^21^

Considering the strong connection between the TREM-1 and TLR4 signaling pathways, the effect of TLR4 activation on NET production, and the deleterious role of NETs in the early phase of sepsis by promoting endothelial activation and dysfunction, we hypothesized that TREM-1 plays a role in NET generation and/or potentiates the deleterious effects of NETs. Therefore, we investigated the effects of TREM-1 activation on NET production and NET-mediated endothelial cell activation and vascular dysfunction. This study reveals a novel mechanism of NETosis during sepsis.

## Materials and methods

All experiments involving animals were approved by our Institutional Animal Care and Use Committee (number 01079.01) and conducted according to the guidelines from Directive 2010/63/EU of the European Parliament on the protection of animals used for scientific purposes.

### Animals

Eight-week-old male *Trem1*-knockout (*Trem1*^*−/−*^ or *Trem1-*ko) and C57Bl/6 wild-type littermate (WT-L) mice weighing 22 to 25 g were used. *Trem1*^*−/−*^ mice were generated by engineering a deletion in exon 1 of the *Trem1* gene. Animals were purchased from Janvier Labs (Le Genest-Saint-Isle, France). The mice were housed in ventilated cages maintained at constant temperature (21 ± 1 °C) and humidity (55 ± 10%) in a 12-h light/dark cycle-controlled room. The mice were allowed free access to standard mouse chow and water. All mice were housed under specific and opportunistic pathogen-free conditions until the start of the experiment.

### Blood collection

Whole blood from healthy male donors aged 18–70 years old was obtained from the French blood agency (Etablissement Francais du Sang EFS) under the agreement ALC/PIL/DIR/AJR/FO/6060. All donors signed an informed consent form.

### TREM-1 inhibitory peptides

To assess the effects of specific TREM-1 inhibition on human cells and in mice, we used species-specific analogs of the LR12 peptide: human LR12 (hLR12: LQEEDAGEYGCM) and murine LR12 (mLR12: LQEEDTGEYGCV). LR12 is a specific functional inhibitor of TREM-1 that is designed to mimic a highly conserved sequence across various species. There is a difference of two amino acids between murine and human LR12, and this difference confers species specificity (Supplementary Figs. 1 and 2). These peptides were chemically synthesized (Pepscan Presto BV, Lelystad, The Netherlands) as COOH terminally amidated peptides with >95% purity, as confirmed by mass spectrometry and analytic reverse-phase high-performance liquid chromatography. These peptides were free of endotoxin.

### Mouse model of endotoxemia

The mice were administered 15 mg/kg lipopolysaccharide (LPS; *Escherichia coli* serotype O127:B8; Sigma Chemical, St. Louis, France), or an equivalent volume of saline intraperitoneally was randomly divided into four groups and received a single intraperitoneal (i.p.) injection of mLR12 at a dose of 5 mg/kg (this dose had already been used in a previous study with a similar peptide^21^) or an equivalent volume of saline 1 h later. At 6 h, the mice were anesthetized, and blood was sampled by cardiac puncture and organs were collected before the mice were sacrificed. Plasma was obtained after the centrifugation of whole blood (300 × *g*, 10 min) and stored at −80 °C until further analysis. Lungs were harvested to assess markers of extracellular DNA release.

### Human and murine neutrophil isolation

The EasySep™ Direct Human Neutrophil Isolation Kit and EasySep™ Mouse Neutrophil Enrichment Kit (STEMCELL, Grenoble, France) were used to collect neutrophils from the peripheral blood of healthy donors and bone marrow from the femurs of WT-L and *Trem1*^*−/−*^ mice, respectively, according to the manufacturer recommendations. After isolation, human or murine neutrophils were suspended in phenol red-free RPMI-1640 (Thermo Fisher, Illkirch-Graffenstaden, France) supplemented with 2% fetal calf serum. The purity of the neutrophil preparations was analyzed by flow cytometry (all >85%), and a TC10 automated cell counter with trypan blue from Bio-Rad was used to assess cell viability (always >95%).

### Time course of DNA release

Freshly isolated human neutrophils were immediately plated on 96-well black plates (2 × 10^5^ cells/well) in the presence of 1 μM SYTOX Green (Invitrogen, Saint Aubin, France), a noncell-permeant DNA binding dye. Cells were then stimulated with increasing concentrations of phorbol 12-myristate 13-acetate (PMA) (10, 50, or 100 nM) (Sigma-Aldrich, Lyon, France) or LPS (0.1, 0.5, 1, or 10 μg/ml) (Sigma-Aldrich, Lyon, France) at 37 °C in 5% CO_2_ in the dark. Extracellular DNA release was examined by measuring the green fluorescence at 0, 60, 120, 180, 240 min and 6 h with a microplate fluorescence reader at an excitation wavelength of 485 nm and an emission wavelength of 527 nm (Multilabel Counter VICTOR-3, PerkinElmer). These experiments allowed us to determine the optimal concentration of each stimulus, as well as the time of maximal NET release. All conditions were repeated six times.

### NET release

Human neutrophils were isolated as previously described and stimulated with LPS (10 μg/ml) or PMA (50 nM) for different time periods (0.5, 1, 2, 3, 4, or 6 h) with or without hLR12 (25 or 50 mg/ml) coincubation.

Neutrophils were isolated from WT-L and *Trem1*-ko mice, stimulated with LPS (10 μg/ml) or PMA (50 nM) and incubated with mLR12 (25 mg/ml) for 3 h.

All cells were incubated at 37 °C in 5% CO_*2*_ in the dark.

A single concentration of the agonist anti-TREM-1 (αTREM-1) (MAB1278, Biotechne, R&D Systems, USA) (10 μg/ml) was administered to LPS-stimulated human neutrophils to evaluate the effect on NET release. DNA generation was examined by measuring the green fluorescence as previously described. An isotype-matched antibody was used as a negative control.

### NET collection

NETs from LPS-activated neutrophils were collected as previously described.^31^ Briefly, freshly isolated human or murine neutrophils were plated in 6-well culture plates (2.5 × 10^6^ cells/ml) and stimulated with LPS (10 μg/ml) for 3 h at 37 °C and 5% CO_2._ The culture medium was then carefully removed, and each well was washed twice with 1 ml of cold phosphate-buffered saline (PBS). The wash solution containing NETs was then centrifuged at 450 × *g* for 10 min to remove the cells. The collected supernatant containing the NETs was further centrifuged at 16,000 × *g* for 10 min to pellet the NETs. After removing the supernatant, the pellet containing the NETs was resuspended in fresh RPMI containing 2% fetal bovine serum (FBS). The isolated NETs were stored at −20 °C until use. NETs were also collected from untreated neutrophils. NETs were analyzed for LPS contamination by a Chromogenic Endotoxin Quant Kit (Thermo Fisher Scientific, Illkirch-Graffenstaden, France) according to the manufacturer’s instructions. We found an acceptably low level of LPS (≤3.37 EU/ml) in the collected NETs.

### Quantification of cell-free DNA and MPO-DNA complexes

A Quant-iT PicoGreen Double-stranded DNA Assay Kit (Invitrogen, Paris, France) was used to quantify NETs in cell supernatants or plasma according to the manufacturer’s instructions.

Murine plasma MPO-DNA was quantified as previously described.^15^ Briefly, a 96-well plate (Thermo Fisher Scientific, Illkirch-Graffenstaden, France) was coated with the capture antibody (anti-mouse MPO, #07-496, Merck Millipore, France) (5 µg/ml) or murine isotype control (IgG, 5 µg/ml) overnight at 4 °C. After washing the plate three times (300 μl each), 20 μl of sample and 80 μl of incubation buffer containing a peroxidase-labeled anti-DNA mAb (Cell Death ELISAPLUS, Roche; dilution 1:25) were added to the wells. The plate was incubated for 2 h at room temperature with shaking at 300 r.p.m. After three washes (300 μl each), 100 μl of peroxidase substrate (ABTS) was added. The absorbance at a wavelength of 405 nm was measured after 20 min of incubation at room temperature in the dark.

### Cell culture and stimulation

Human pulmonary microvascular endothelial cells (HPMECs) were purchased from PromoCell (six different batches originating from six different donors) (Heidelberg, Germany). After the cells were thawed according to the provider recommendations, the cells were maintained in complete endothelial cell growth medium MV (PromoCell) at 37 °C in a 5% CO_2_ humidified atmosphere incubator. All experiments were performed using cells at passages 2 or 3. Endothelial cells were stimulated with 10 μg of isolated NETs in the presence or absence of 25 μg/ml hLR12 for 6 h.

### Flow cytometry analysis

HPMECs were subjected to flow cytometry to assess the expression of endothelial activation/dysfunction markers (vascular cell adhesion molecule-1 (VCAM-1), intercellular adhesion molecule-1 (ICAM-1), E-selectin, and TREM-1) following 6 h of stimulation with NETs. Endothelial cells were detached using trypsin and centrifuged at 400 × *g* for 5 min. The cells were then incubated with different labeled antibodies (TREM-1-FITC, Vcam-1-PE, E-selectin-PE, or Icam-1-PerCP, Miltenyi Biotech) or corresponding isotype controls for 30 min at 4 °C in the dark. After being washed twice with PBS, the cells were resuspended and fixed in 4% paraformaldehyde. An Accuri C6 flow cytometer (Becton Dickinson, San Jose, CA, USA) was used for analysis.

### Vascular reactivity

Healthy mice were sacrificed by pentobarbital administration (100 mg/kg i.p.), and the thoracic aorta and mesenteric arteries were immediately collected. The vessels were placed in 6-well culture plates with RPMI and incubated with 10 μg of isolated mouse NETs with or without mLR12 (25 mg/ml). Three hours after stimulation, the vessels were placed in an ice-cold physiological salt solution composed of 130 mM NaCl, 14.9 mM NaHCO_3_, 3.7 mM KCl, 1.2 mM MgSO_4_, 2.5 mM CaCl_2_, 1.2 mM KH_2_PO_4_, and 5.5 mM glucose. Two-millimeter-long rings were cut and mounted on a 40 μm stainless-steel wire in a small vessel myograph (EMKA Technologies, France). The preparation was supplied with a carbogen gas mixture (95% O_2_, 5% CO_2_). After an equilibration period (at least 20 min) under optimal passive tension, two successive contractions in response to the combination of KCl depolarization and phenylephrine (Sigma, St. Louis, MO, USA) were used to measure the maximal contractile capacity of the vessels. Concentration–response curves to phenylephrine (1 nM to 100 μM) were obtained. After a washout period of 20 min, the vessels were precontracted with phenylephrine (1 μM) to 80% of the maximum contraction, and the acetylcholine (Sigma, St. Louis, MO, USA) concentration–response curve was obtained (1 nM to 100 μM).

### Protein isolation and western blot analysis

Western blot analyses were performed on proteins in human neutrophils and NETs and the aortas, mesenteric arteries, and lungs of mice.

NET proteins were isolated by using the MNase method. In brief, 2.5 × 10^6^ human neutrophils were plated in 6-well culture plates in RPMI. The cells were treated with LPS or LPS/hLR12 for 3 h at 37 °C in 5% CO_2_. NETs were then digested with 10 U/ml MNase-1 for 20 min at 37 °C. Ethylenediaminetetraacetic acid (5 mM) was used to stop MNase activity. The samples were then centrifuged at 300 × *g* to remove whole cells and then centrifuged again at 16,000 × *g*. Proteins were isolated by precipitation after the addition of acetone overnight at −20 °C, followed by centrifugation at 14,000 × *g*.

To isolate neutrophil proteins, human neutrophils were stimulated with LPS (10 μg/ml) alone or in the presence of a TREM-1 agonist for different times (2, 10, 30, 60, and 180 min). Proteins were isolated by using RIPA buffer.

To isolate tissue proteins, aortas, mesenteric arteries, and lungs were lysed in PhosphoSafe Extraction Reagent (Novagen, Merck Biosciences, Nottingham, UK) and centrifuged for 5 min at 16,000 × *g* at 4 °C to collect the supernatant.

Protein concentrations were determined using a BCA Protein Assay Kit (Pierce; Thermo Scientific). Thirty micrograms of each sample was electrophoresed on a Criterion XT Bis-Tris Gel 4–12% (Bio-Rad) and transferred to a polyvinylidene difluoride membrane (Millipore, Saint-Quentin-en-Yvelines, France). Membranes were blocked with 5% w/v skim milk powder in TBST (0.1 M Tris-HCl, pH 8, 1.5 M NaCl, and 1% Tween-20) for 2 h at room temperature and subsequently incubated with primary antibodies overnight at 4 °C. The following primary antibodies were used: spleen tyrosine kinase (SYK), phospholipase Cγ (PLCγ) (Cell Signaling, Saint-Quentin-en-Yvelines, France), peptidylarginine deiminase 4 (PAD4), Cit-H3 (Abcam, France), TREM-1 (Bio-Rad, France), inducible nitric oxide synthase (iNOS) and cyclo-oxygenase-2 (COX-2) (Cell Signaling, Saint-Quentin-en-Yvelines, France).

After being vigorously washed in TBST, the membranes were incubated with secondary antibodies conjugated to horseradish peroxidase for 1 h at room temperature. Immunocomplexes were detected with SuperSignal West Femto Substrate (Pierce; Thermo Scientific). Actin (Cell Signaling Technology) was used for normalization (as an internal control). Data acquisition and quantitative signal density analyses were performed by a LAS-4000 imager (FSVT) and Multi-Gauge software (LifeScience Fujifilm, Tokyo, Japan).

### Confocal microscopy

Isolated human neutrophils were plated and stimulated on Nunc LabTek chambers (Thermo Fisher Scientific, Waltham, MA, USA) with LPS (10 µg/ml) for 3 h. After stimulation, the cells were then washed and fixed with paraformaldehyde (4%) for 20 min and blocked in 1% bovine serum albumin for 1 h before being incubated with anti-Cit-H3 (Abcam) and anti-TREM-1 (Bioss), while DNA was stained with TO-PRO-3 at 4 °C overnight. After being washed with PBS, the coverslips were mounted with Vectashield (Vector Laboratories, CA, USA) and visualized through sequential scanning on an SP5 confocal microscope (Leica, Wetzlar, Germany). Images were processed using LAS-AF Lite software (Leica, Wetzlar, Germany).

### ROS generation

The production of intracellular ROS by resting and LPS-stimulated human neutrophils was measured using the ROS-specific probe dichlorodihydrofluorescein diacetate and acetyl ester (H_2_DCFDA) (Thermo Fisher Scientific, Illkirch-Graffenstaden, France) and flow cytometry. Human neutrophils were suspended at 10^6^ cells/ml in RPMI with 10% FBS and were coincubated with 1 μM H_2_DCFDA and stimulation for 1 h at 37 °C in a 5% CO_2_ atmosphere. Then, the samples were analyzed by flow cytometry (BD Accuri C6 flow cytometer from BD Biosciences) with an excitation wavelength of 488 nm and an emission wavelength of 525 nm.

### Intracellular calcium measurement

Calcium analysis was performed using flow cytometry (C6 Accuri, BD). Human primary neutrophils were stimulated with LPS (100 ng/ml) for 3 h, washed and incubated with pluronic acid and fluo-3-acetoxymethyl ester (10 µM; Sigma-Aldrich, France) for 30 min in Ringer’s solution (145 mM NaCl, 5.4 mM KCl, 2 mM CaCl_2_, 1 mM MgCl_2_, 10 mM glucose, 10 mM HEPES, and 0.1% bovine serum albumin, pH adjusted to 7.5). After baseline acquisition for 1 min, intracellular calcium mobilization was triggered by stimulation with the TREM-1 agonist, and the cells were monitored for 3 min. The results were normalized between the baseline and the maximal signal induced by ionomycin during the last minute of acquisition (1 µM; Sigma-Aldrich, France).

### Data presentation and statistical analyses

Statistical analyses were performed using GraphPad Prism 7. Between-group differences were analyzed by one-way analysis of variance (ANOVA) with Dunnett’s and Tukey’s post hoc tests, two-way ANOVA with Bonferroni post hoc test, or *t* tests. A *p* value of <0.05 was considered to represent a statistically significant difference. All data are presented as the mean ± SD.

## Results

### TREM-1 activation potentiates NET generation

**Fig. 1:**
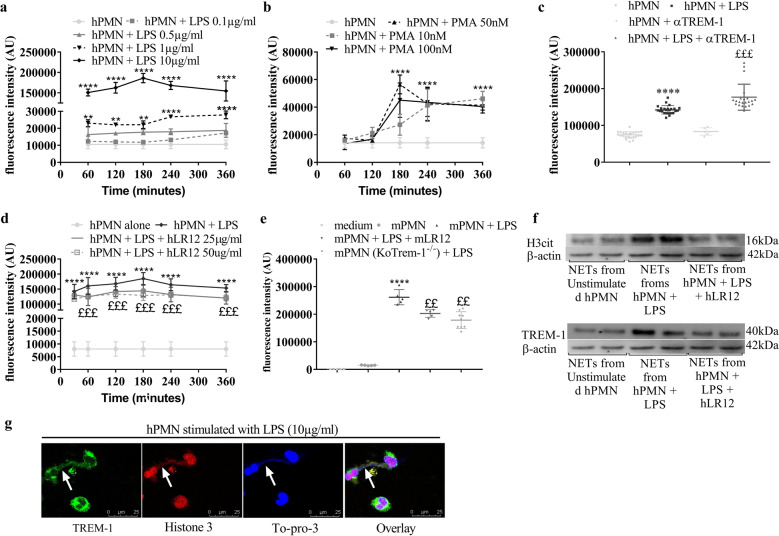
LPS and PMA induce NETosis in a dose- and time-dependent manner, and TREM-1 activation potentiates LPS-induced NET generation. NETosis, as assessed by SYTOX Green fluorescence intensity as a proxy for DNA release, was induced by LPS (0.1, 0.5, 1, and 10 µg/ml) or PMA (10, 50, and 10 nM) in a dose- and time-dependent manner (**a**, **b**). TREM-1 activation in human neutrophils induced by 3 h of coincubation with the monoclonal agonist antibody (αTREM-1) alone (10 µg/ml) or with LPS and αTREM-1 (10 µg/ml) increased NETosis (**c**), while TREM-1 inhibition by hLR12 decreased NETosis (**d**). Murine neutrophils were stimulated with LPS for 3 h to induce NETosis, which was reduced in neutrophils isolated from *Trem1*-knockout mice or in cells treated with mLR12 (**e**). NETs isolated from LPS-stimulated human neutrophils expressed significantly more Cit-H3 and TREM-1 than NETs isolated from unstimulated neutrophils, and 25 µg/ml hLR12 significantly decreased these expression levels (**f**). * or ^£^*P* < 0.05, ** or ^££^*p* < 0.01, *** or ^£££^*p* < 0.005, **** or ^££££^*p* < 0.005 (* versus PMN alone, ^£^versus LPS); one- and two-way ANOVA with Tukey’s test and *t* tests were used; *n* = 5–12). Confocal microscopy (SP5) analysis of unstimulated and LPS-stimulated human neutrophils stained for H3-cit, TREM-1, and TO-PRO-3 (*n* = 3; TREM-1: green; histone 3: red; TO-PRO-3: blue and merged) (scale bars, 25 µm) (**g**). White arrow heads indicate web-like scaffolds of extracellular DNA stained with different markers (TREM-1, Histone 3 and To-pro-3)

We first determined the ideal concentration and time to induce maximal NET release from human neutrophils. Although low doses of LPS had no or weak effects, 10 μg/ml LPS robustly induced NET formation, with a maximum at 3 h (Fig. 1a). A similar pattern was observed in the presence of PMA (Fig. 1b), although NET formation remained lower than that observed upon LPS stimulation. We next examined the effect of TREM-1 activation on LPS-mediated NETosis. Human neutrophils were stimulated with αTREM-1 (10 μg/ml) with or without LPS (10 μg/ml) for 3 h, and while αTREM-1 alone elicited a very weak response, αTREM-1 potentiated LPS-induced NET release (Fig. 1c). In contrast, inhibiting TREM-1 activation through the use of the inhibitory peptide hLR12 reduced LPS-induced NET production (Fig. 1d). To exclude the potential off-target effects of LR12 or αTREM-1, we investigated NETosis in murine neutrophils. Pharmacologic inhibition of TREM-1 (with mLR12), as well as genetic ablation (*Trem1*-ko), resulted in reduced LPS-induced NET production (Fig. 1e). hLR12 alone did not affect NETosis (data not shown). Western blot analysis of citrullinated histone H3 (Cit-H3) confirmed the hLR12-induced reduction in LPS-mediated NETosis in human neutrophils (Fig. 1f). We finally examined whether TREM-1 was a component of NETs. Western blot analysis of isolated NETs revealed the presence of TREM-1 (Fig. 1f). This result was confirmed by confocal microscopy: LPS-induced NETs were observed as web-like scaffolds of extracellular DNA decorated with extracellular Cit-H3 and TREM-1 (Fig. 1g).

Taken together, these data suggest the involvement of TREM-1 activation during NETosis, as well as the participation of TREM-1 in the NET structure.

### TREM-1 inhibition decreases NET-induced endothelial cell activation

**Fig. 2:**
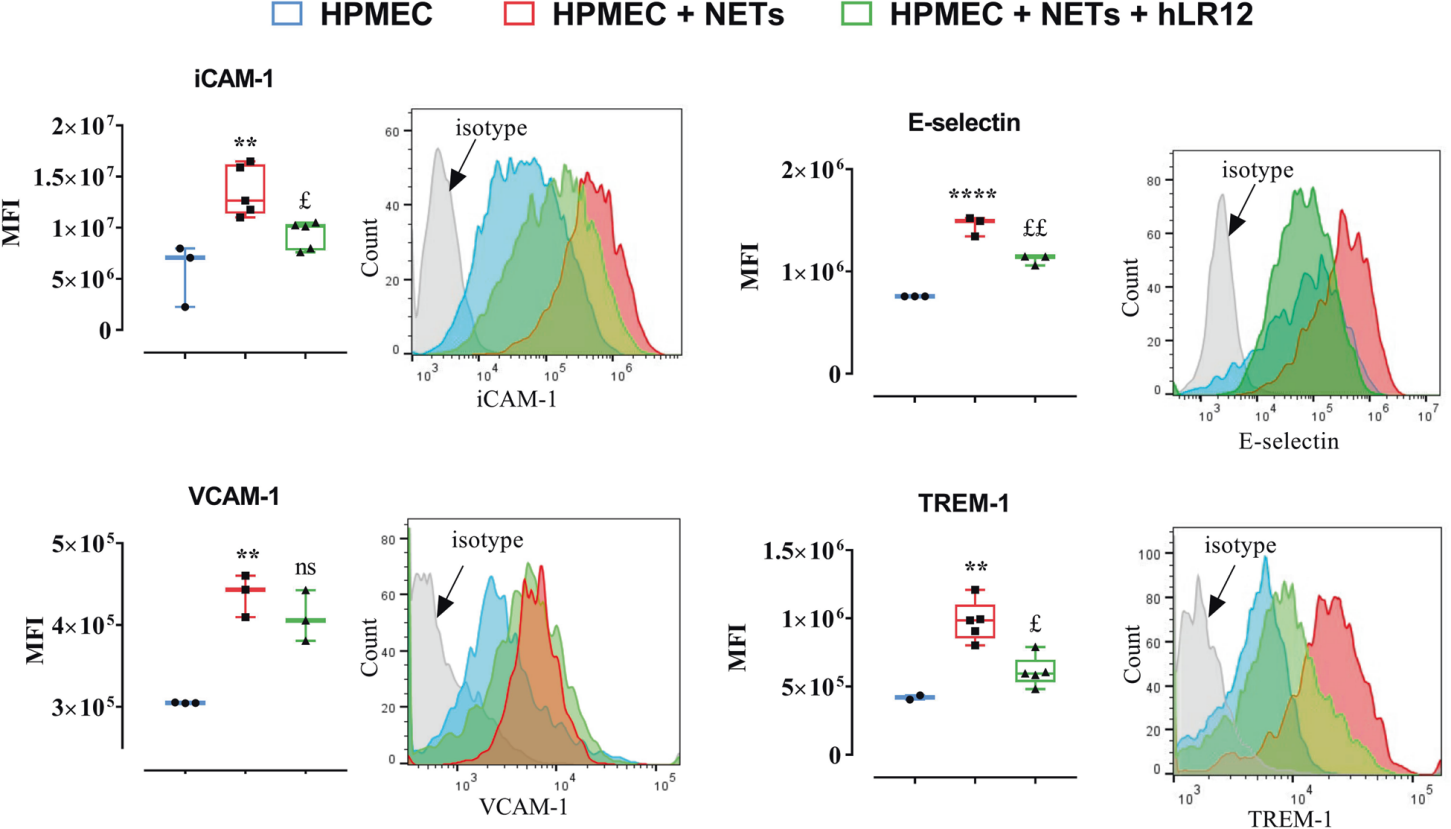
TREM-1 inhibition decreases NET-induced endothelial cell activation. The expression of several endothelial activation markers was determined by flow cytometry 6 h after the coincubation of HPMECs with NETs (10 µg/ml) and hLR12 (25 µg/ml). n.s. Not significant, * or ^£^*P* < 0.05, ** or ^££^*p* < 0.01, *** or ^£££^*p* < 0.005, **** or ^££££^*p* < 0.005 (* versus PMN alone, ^£^ versus LPS). The data were analyzed by one-way ANOVA with Tukey’s test; *n* = 4)

We examined whether isolated NETs (from LPS-activated human neutrophils) could activate endothelial cells. HPMECs were stimulated for 6 h with NETs (10 µg/ml) with or without hLR12 coincubation. Fluorescence-activated cell sorting analysis showed that NETs upregulated ICAM-1, E-selectin, VCAM-1, and TREM-1 expression. This upregulation was partly prevented by hLR12 (Fig. 2), except that of VCAM-1 (Fig. 2). In contrast, hLR12 alone had no effect on HPMECs (data not shown).

These results indicate that NETs are able to activate endothelial cells in a TREM-1-dependent manner because TREM-1 inhibition reduces endothelial cell activation.

### TREM-1 inhibition reduces NET-induced vascular dysfunction

**Fig. 3:**
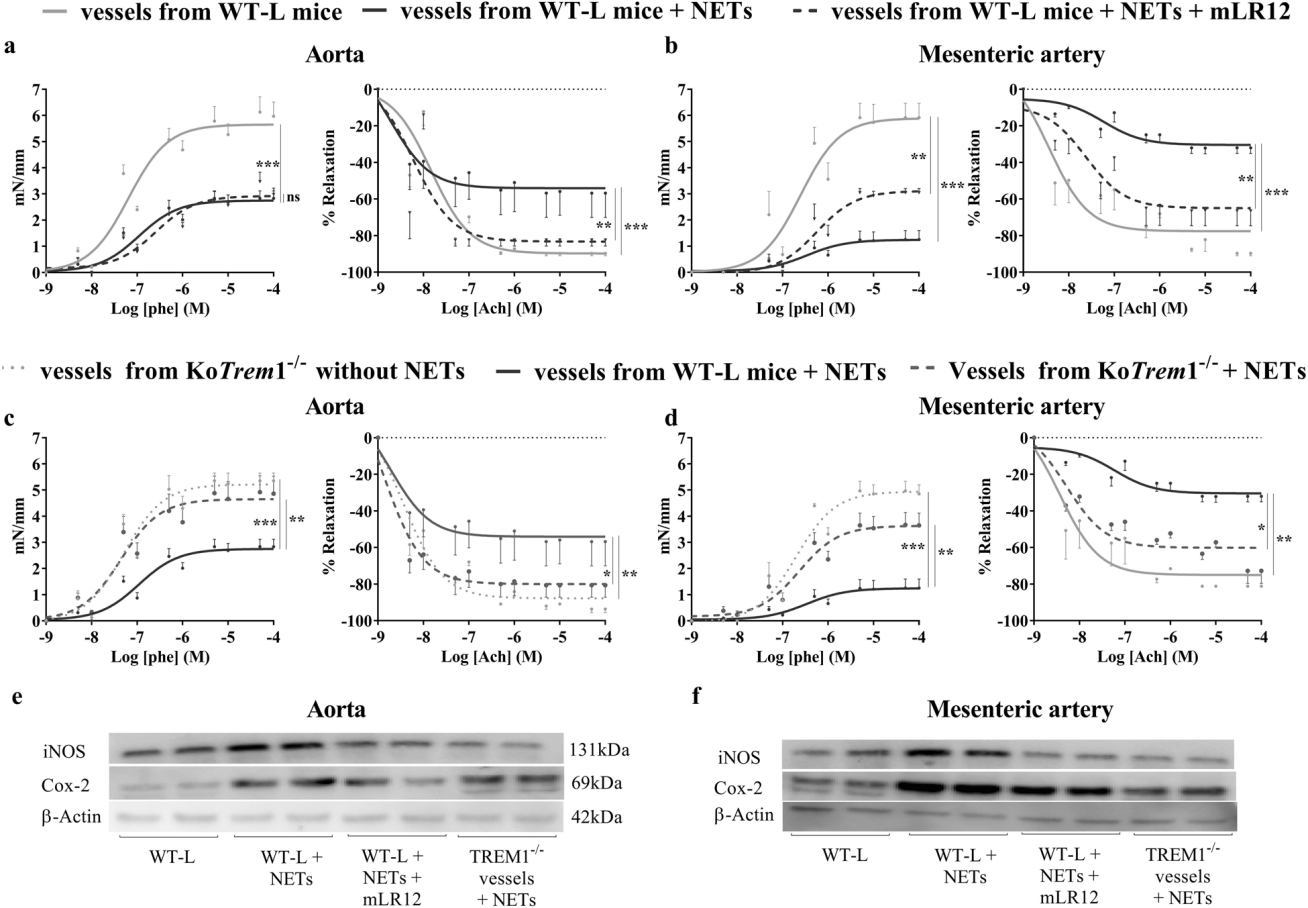
TREM-1 inhibition dampens NET-induced vascular dysfunction. Concentration–response curves to phenylephrine (Phe) and acetylcholine (Ach) in murine aortas and mesenteric arteries incubated or without NETs (10 µg/ml) and hLR12 (25 µg/ml) for 3 h (**a**, **b**). Vessels from *Trem1*-knockout mice were incubated or without NETs (10 µg/l) (**c**, **d**). n.s. Not significant, *p < 0.05, **p < 0.01, ***p < 0.005, *n* = 4–5 per group (two-way ANOVA). Western blots showing iNOS and COX-2 expression in the aorta and mesenteric arteries (**e**, **f**)

We next examined the effect of NETs on vascular function and the impact of the pharmacological inhibition or genetic ablation of TREM-1. To assess vascular reactivity, aortas and mesenteric arteries from WT-L and *Trem1*-ko mice were harvested and incubated with NETs isolated from LPS-activated murine neutrophils. NETs profoundly impaired the contractility and relaxation of both types of vessels (Fig. 3a, b). While mLR12 showed little effect on NET-induced contractility impairment, it almost completely restored endothelium-dependent relaxation. Furthermore, *Trem1* genetic deletion also protected against NET-induced vascular dysfunction (Fig. 3c, d).

The overexpression of iNOS and COX-2 is crucial in mediating vascular dysfunction.^32,33^ Western blot analysis revealed that NETs upregulated the expression of these two proteins in both aortas and mesenteric arteries, and this upregulation was prevented by genetic or pharmacologic TREM-1 inhibition (Fig. 3e, f).

### Pharmacological inhibition of TREM-1 reduces NET release in vivo

**Fig. 4:**
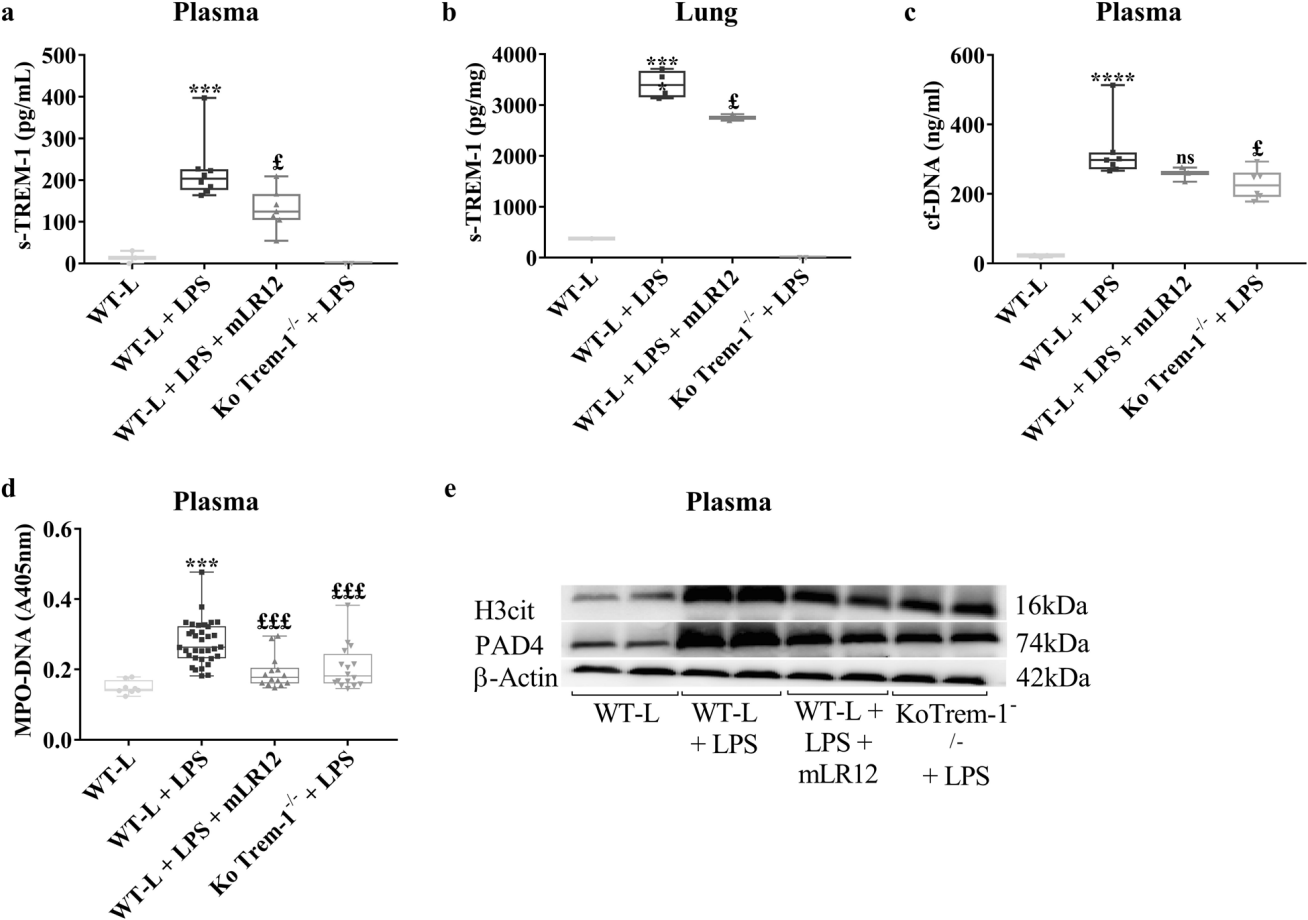
TREM-1 inhibition decreases NET release during experimental LPS-induced septic shock. Mice were administered LPS (15 mg/kg) with or without mLR12 (5 mg/kg), and the following analyses were performed 6 h later: ELISA quantification of sTREM-1 in the plasma (**a**) and lung (**b**) from the control, LPS, LPS/mLR12, and *Trem1*-knockout groups; plasma concentration of cell-free DNA (**c**) and MPO/DNA (**d**) was determined using Quant-iT PicoGreen assays and ELISA, respectively. The data were analyzed by *t* tests and one-way ANOVA. n.s. Nonsignificant, * or ^£^*p* < 0.05, ** or ^££^*p* < 0.01, *** or ^£££^*p* < 0.001 (* versus control, ^£^ versus LPS). Western blot analysis of proteins extracted from the lung were analyzed with Abs against H3-cit and PAD4 (**e**). Graphs are plotted as the mean ± SD (*n* = 4–6 per group)

As previously described,^34^ we observed that the concentration of the soluble form of TREM-1 was increased in both plasma and lung homogenates 6 h after LPS injection (Fig. 4a, b). To assess NET release in the plasma of septic animals, we measured both circulating cell-free DNA (Fig. 4c) and MPO-DNA (Fig. 4d). As the lung is a major target organ during sepsis, we also indirectly determined pulmonary NET formation by measuring Cit-H3 and PAD4 expression in lung homogenates (Fig. 4e). Of note, Cit-H3 and PAD4 have been suggested to be markers of sepsis severity.^35–37^ While LPS increased NET release in the plasma and lungs, this effect was prevented by TREM-1 inhibition.

### TREM-1 is involved in key steps of NETosis

**Fig. 5:**
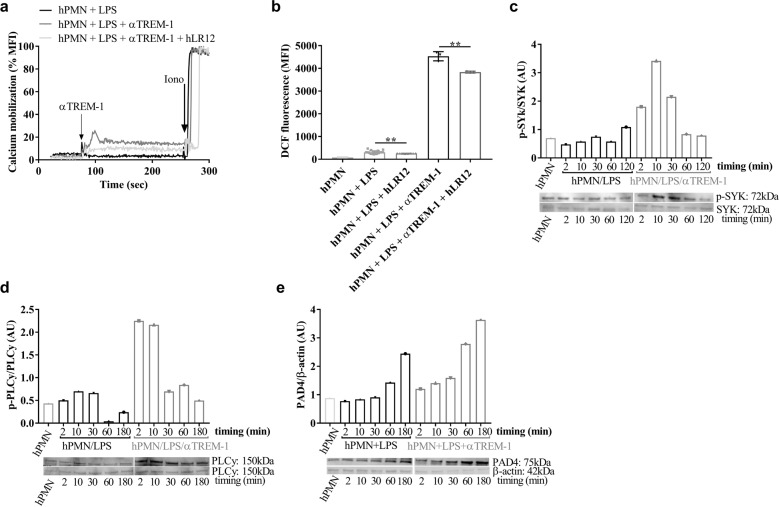
TREM-1 inhibition impairs key steps of NETosis. Kinetics of intracellular calcium release induced by LPS (100 ng/ml) and αTREM-1 (10 µg/ml) with or without hLR12 (25 µg/ml) were monitored for 300 s; 100% calcium release was achieved with the addition of ionomycin at the end of the assay (**a**). Intracellular ROS production after 2 h of incubation with LPS (100 ng/ml) or coincubation with αTREM-1 (10 µg/ml) with or without hLR12 (25 µg/ml) (**b**). Western blot analysis of proteins extracted from primary human neutrophil cells stimulated for 2, 10, 30, 60, and 180 min with LPS alone (10 µg/ml) or with αTREM-1 (10 µg/ml) at the indicated times after 2 h of pretreatment with LPS and analyzed with Abs against phospho-SYK (p-SYK), SYK (**c**), phospho-PLCγ (p-PLCγ), PLCγ (**d**), PAD4 (**e**), and β-actin (as a reference protein). The data are representative of at least three different experiments. *P < 0.05, **p < 0.01, and ***p < 0.001 as determined by two-tailed Student’s *t* tests. LPS and LPS/αTREM-1 were compared between times (2 min LPS versus 2 min LPS/αTREM-1)

NETosis mechanisms involve several key steps, including calcium flux,^38^ ROS generation,^39^ and the phosphorylation of PLCγ,^40^ ERK1/2,^28^ and SYK.^41^ To examine how TREM-1 pathway activation increases LPS-induced NETosis, we assessed the release of intracellular calcium using fluo-3-loaded human primary neutrophils and flow cytometry. As previously described,^32^ TREM-1 activation induced intracellular calcium release in LPS-activated neutrophils (Fig. 5a), and the hLR12 decreased this effect. As a control and to normalize the results, we used thapsigargin, which increases the cytosolic calcium concentration by inhibiting the SERCA (sarcoendoplasmic reticulum calcium transport ATPase) pump (data not shown), and ionomycin as an ionophore to induce a maximal response. The same response profile was also observed when examining ROS production (Fig. 5b). These results indicate that activated TREM-1 induces intracellular calcium flux and ROS production and that TREM-1 inhibition reduces these effects. To further verify the involvement of TREM-1 pathway activation in LPS-induced NETosis, we quantified p-SYK/SYK, p-PLCγ/PLCγ, and PAD4 by western blotting. We observed increased phosphorylation of SYK and PLCγ in conditions in which TREM-1 crosslinking was induced by incubation with αTREM-1 compared to LPS alone (Fig. 5c, d). αTREM-1 also increased PAD4 expression in LPS-activated neutrophils (Fig. 5e). These results confirm that TREM-1 activation potentiates the activation of key pathways implicated in NETosis following TLR4 engagement.

## Discussion

Due to its key role as an amplifier of the innate immune response that interacts with a broad set of TLRs,^20,25–27,33,35–37,42^ TREM-1 is emerging as a ubiquitous regulator of many pathological processes, especially septic shock. The TREM-1 inhibitory peptide LR12, also called nangibotide under its international nonproprietary name, has been shown to be well tolerated by septic shock patients in a clinical phase 2a trial.^43^ More recently, phase 2b trial has been initiated, and recruitment is ongoing (NCT04055909).

The present study is the first to investigate the direct relationship between TLRs and TREM-1 activation in NETosis. The rapid deployment of neutrophil effector functions is a key mechanism by which the innate immune system mounts a rapid and efficient response against infection. TREM-1 ligation on primary human neutrophils by the use of an agonistic anti-TREM-1 antibody has been shown to induce the activation of intracellular pathways, particularly AKT, STAT5, p38, and ERK1/2,^25^ TREM-1-dependent degranulation, intracellular calcium flux, and ROS release.^37^ Compared to monocytes, neutrophils express high levels of TREM-1 at the membrane, allowing rapid activation of the TREM-1 pathway.^32^ TREM-1-dependent intracellular calcium flux, ROS release, and the production of interleukin-8 (IL-8) by human neutrophils may depend on the coactivation of TREM-1 and TLR4.^26,32^ Fortin et al.^25^ demonstrated that TREM-1 was quickly mobilized at the membrane into GM1 lipid rafts following TLR4 engagement and colocalized with TLR4, which was suggested to induce the formation of an innateosome at the membrane of innate immune cells to rapidly and efficiently respond to invading microorganisms. In line with these results, we previously described a TLR-dependent mechanism by which TREM-1 clustering and oligomerization at the membrane was a key molecular step in receptor activation.^32^ Similarly, in the present study, the ligation of TREM-1 alone by the agonistic anti-TREM-1 antibody was not associated with NET release by human polymorphonuclear leukocytes (PMNs) and required coactivation of TLR4.

TREM-1-TLR crosstalk is known to potentiate various neutrophil effector functions, such as cytokine and ROS release,^26^ and we confirmed here that TREM-1 activation also impacted TLR-induced NET release. Indeed, coactivation of TREM-1 and TLR4 resulted in an increase in NET release by human primary neutrophils. Blocking TREM-1 activation by species analogs of the LR12 peptide was associated with a decrease in TLR-induced NET release by human and murine neutrophils. A similar effect was observed in PMNs from TREM-1-KO mice. These results confirm that there is functional crosstalk and a synergistic effect of TREM-1 activation on TLR-induced NET release by PMNs.

The crosstalk between neutrophils and endothelial cells plays a critical role in mounting an inflammatory response during septic shock, and NETs are important participants in vascular inflammation.^9,12,44,45^ Indeed, it has been shown that NET structures are made of chromatin decorated with several proteins, such as histones and myeloperoxidase,^4^ and that NETs induce endothelial cell activation, as evidenced by high expression of adhesion molecules and increase leukocyte adhesion to cell monolayers,^13^ as well as cellular dysfunction.^45–47^ In particular, histones directly interact with TLR2 and TLR4 to induce cell signaling via MyD88, MAPK, and NF-κB and cytokine production.^48^ To investigate the role of TREM-1 in this process, we collected NETs from stimulated neutrophils and analyzed their effects on endothelial cell activation and vascular reactivity in the presence of the TREM-1 inhibitory peptide LR12. We observed a NET-induced increase in ICAM-1, E-selectin, VCAM-1, and TREM-1 expression on HPMECs that was inhibited by the addition of LR12. This finding suggests a role for TREM-1 in the NET-induced activation of endothelial cells, which may also impact vascular function.

Indeed, NETs impaired vasoreactivity, vascular relaxation, and vascular contraction, and again, TREM-1 seemed to be implicated in these processes. Pharmacological inhibition of TREM-1 with murine LR12 preserved endothelium-dependent acetylcholine-induced vasodilation. By contrast, TREM-1 inhibition did not affect contractility, largely independent of the endothelium. However, genetic ablation of *Trem1* (*Trem1*-ko) prevented both effects. As previously described,^21^ this protective effect may stem from reduced induction of iNOS and COX-2, two crucial proteins involved in vascular dysfunction. Whether these effects are mediated specifically by TLRs or whether NETs are able to directly activate TREM-1 has not been investigated here and may be of interest to explore, especially considering that we found the presence of TREM-1 on NETs by immunostaining.

Cell-free plasma DNA concentrations have been shown to be higher in ICU nonsurvivors and correlate with lactate levels and sequential organ failure assessment scores.^49,50^ To confirm the implication of TREM-1 in NET release in vivo, we measured both plasma cf-DNA and the cf-DNA associated with MPO (cf-DNA/MPO), which are two major constituents of NET structures, in mice with LPS-induced experimental septic shock. As previously shown,^51^ circulating NETs were abundant after LPS administration. The same held true when we examined NET production by measuring Cit-H3 and PAD4 in the lungs. We found that pharmacological inhibition of TREM-1 by the LR12 peptide largely prevented NET production in this experimental model.

We further confirmed that TREM-1 activation potentiated the activation of key pathways implicated in NETosis. Recent reports identified several key steps of NETosis, including calcium flux,^38^ the generation of ROS,^39^ and the activation of different proteins, such as PLCγ^40^ and SYK,^29,31^ leading to PAD4 and Cit-H3 activation. Our data show that TREM-1 activation potentiates all of these steps in vitro and in vivo, further reinforcing its key role during NETosis.

## Conclusion

Taken together, our findings demonstrate for the first time that TREM-1 activation potentiates NET release and signaling pathways. Moreover, the present study shows that NETs are present in the plasma and lungs of septic mice and can activate endothelial cells and promote vascular dysfunction and that TREM-1 inhibition reduces NET formation and dampens the deleterious effects.

LR12 was previously shown to prevent septic shock-induced organ damage by dampening inflammation, innate immune cell and endothelial dysfunction, and inflammatory cell infiltration.^21,52,53^ We showed here that NET-induced vascular impairment was prevented by blocking TREM-1 with LR12. LR12 administration to LPS-induced mice was associated with a decrease in NET accumulation in the lungs, which has been shown to contribute to tissue damage and organ dysfunction.^54–58^ NETs can activate other cells, such as macrophages and dendritic cells, to produce inflammatory mediators, including IL-1β, tumor necrosis factor-α, and IL-6,^59^ which, in addition to mediating organ damage, can also recruit additional neutrophils to the organs. TREM-1 pharmacological inhibition may thus reduce the increase in NETs that promotes hyperinflammation, thereby decreasing vascular dysfunction, organ injury, and mortality in sepsis. Preventing NET release may thus be a new mechanism of action of LR12.

## Supplementary information

Supplementary Figure S1

Supplementary Figure S2
