# Supplementary figures and images for: Potentiation of NETs release is novel characteristic of TREM-1 activation and the pharmacological inhibition of TREM-1 could prevent from the deleterious consequences of NETs release in sepsis
Source: Cell Mol Immunol. 2021 Jan 8;18(2):452–60. doi: 10.1038/s41423-020-00591-7 (PMC8026640; doi:10.1038/s41423-020-00591-7)

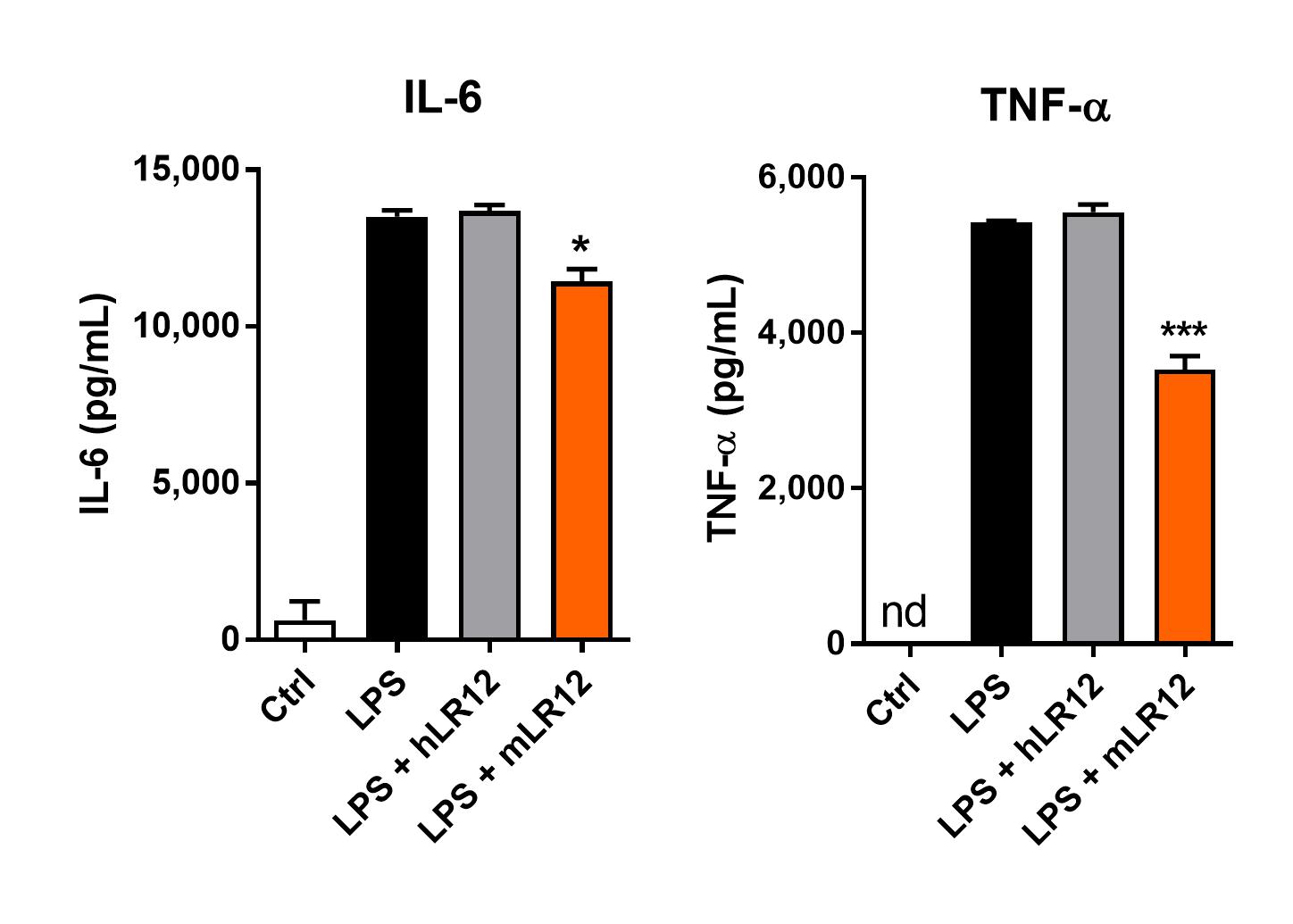

Supplement: Supplementary file 1 — Supplementary Figure S1 [file 41423_2020_591_MOESM1_ESM.jpg]

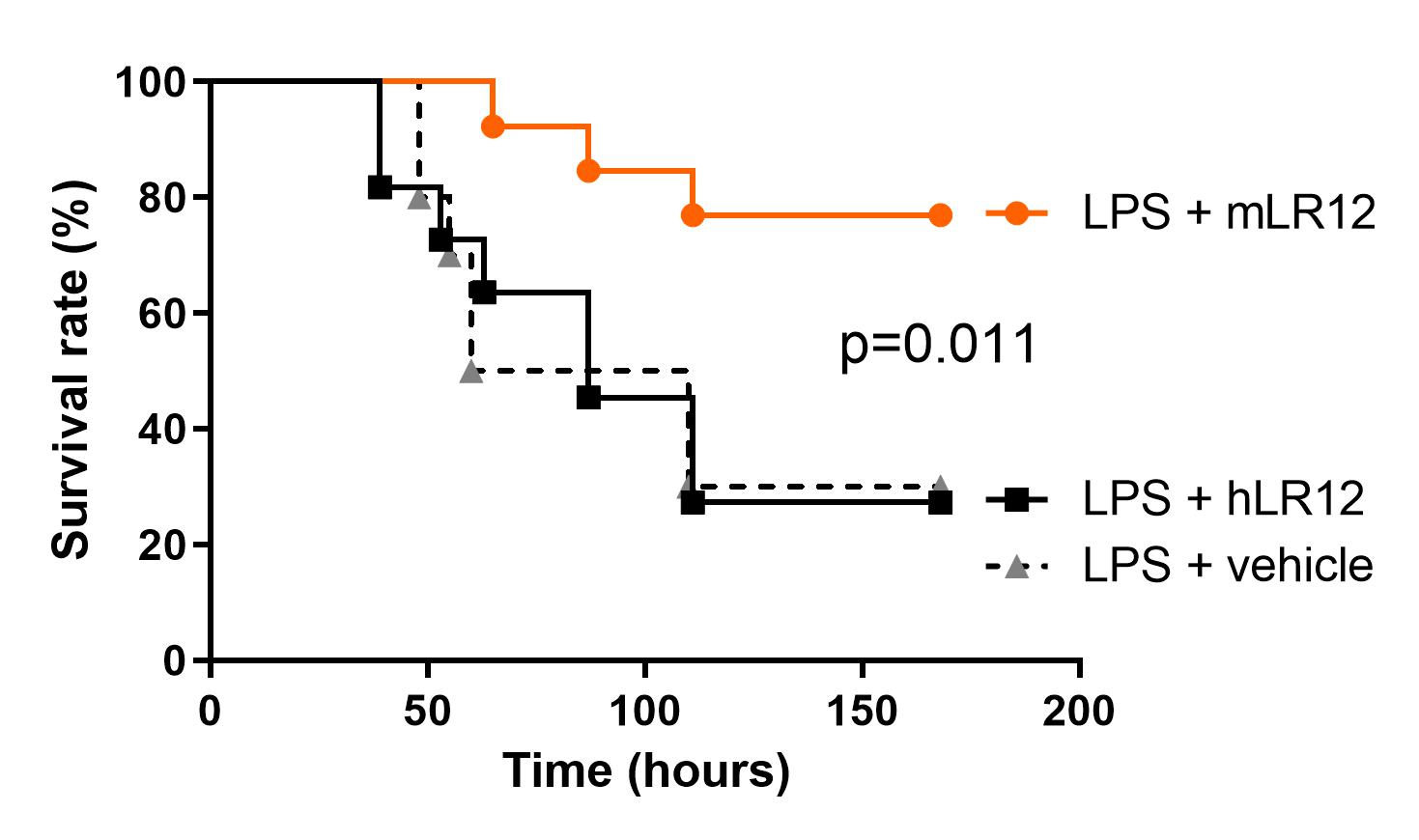

Supplement: Supplementary file 2 — Supplementary Figure S2 [file 41423_2020_591_MOESM2_ESM.jpg]
